# Life-Threatening Primary Varicella Zoster Virus Infection With Hemophagocytic Lymphohistiocytosis-Like Disease in GATA2 Haploinsufficiency Accompanied by Expansion of Double Negative T-Lymphocytes

**DOI:** 10.3389/fimmu.2018.02766

**Published:** 2018-12-03

**Authors:** Seraina Prader, Matthias Felber, Benjamin Volkmer, Johannes Trück, Agnes Schwieger-Briel, Martin Theiler, Lisa Weibel, Sophie Hambleton, Katja Seipel, Stefano Vavassori, Jana Pachlopnik Schmid

**Affiliations:** ^1^Division of Immunology, University Children's Hospital Zurich, Zurich, Switzerland; ^2^Division of Stem Cell Transplantation University Children's Hospital Zurich, Zurich, Switzerland; ^3^Department of Pediatric Dermatology, University Children's Hospital Zurich, Zurich, Switzerland; ^4^Department of Dermatology, University Hospital Zurich, Zurich, Switzerland; ^5^Institute of Cellular Medicine, International Centre for Life, Newcastle University, Newcastle upon Tyne, United Kingdom; ^6^Department for Biomedical Research, University of Bern, Bern, Switzerland; ^7^Pediatric Immunology, University of Zurich, Zurich, Switzerland

**Keywords:** varicella-zoster virus, hemophagocytic lymphohistiocytosis, GATA2 deficiency, primary immumunodeficiencies, NK cell abnormalities, TCRγδ T cells

## Abstract

Two unrelated patients with GATA2-haploinsufficiency developed a hemophagocytic lymphohistiocytosis (HLH)-like disease during a varicella zoster virus (VZV) infection. High copy numbers of VZV were detected in the blood, and the patients were successfully treated with acyclovir and intravenous immunoglobulins. After treatment with corticosteroids for the HLH, both patients made a full recovery. Although the mechanisms leading to this disease constellation have yet to be characterized, we hypothesize that impairment of the immunoregulatory role of NK cells in GATA2-haploinsufficiency may have accentuated the patients' susceptibility to HLH. Expansion of a double negative T-lymphocytic population identified with CyTOF could be a further factor contributing to HLH in these patients. This is the first report of VZV-triggered HLH-like disease in a primary immunodeficiency and the third report of HLH in GATA2-haploinsufficiency. Since HLH was part of the presentation in one of our patients, GATA2-haploinsufficiency represents a potential differential diagnosis in patients presenting with the clinical features of HLH—especially in cases of persisting cytopenia after recovery from HLH.

## Background

Germline mutations leading to haploinsufficiency of Guanin-adenine-thymine-adenine 2 binding protein (GATA2) represent the underlying cause of a disorder encompassing primary immunodeficiency (PID), hematological malignancies and vascular/lymphatic abnormalities (1). The clinical spectrum of GATA2-haploinsufficiency is very variable and ranges from an asymptomatic condition to a potentially life-threatening disease. Typical syndromes related to GATA2-haploinsufficiency include primary lymphoedema with myelodysplasia (Emberger syndrome), monocytopenia and mycobacterial infection (MonoMAC) syndrome, and dendritic cell, monocyte, B and NK lymphoid (DCML) deficiency (2). In addition to unusual infections by atypical mycobacteria, patients may have increased susceptibility to other bacterial or viral pathogens (e.g., human papilloma virus, varicella zoster virus (VZV), and Epstein-Barr virus (EBV)). Patients are also at higher risk of developing hematological malignancies like myelodysplastic syndrome and acute myeloid leukemia. Other disease manifestations include thrombosis, lymphoedema, pulmonary alveolar proteinosis, deafness, and the emergence of solid tumors. Allogeneic hematopoietic stem cell transplantation represents a potentially curative therapy for the hematopoietic and immunologic manifestations in patients with GATA2-haploinsufficiency (3).

Varicella zoster virus is one of eight members of the Herpesviridae family that are human pathogens. Primary VZV infection causes varicella (“chickenpox”) resulting in latent infection, which can reactivate and cause herpes zoster (“shingles”). Although (i) the mechanisms involved in antiviral defenses have not been fully characterized, and (ii) VZV-specific antibodies can prevent primary infection, it appears that cellular immune responses are the most important means of limiting viral replication and altering the course of the disease. The importance of certain cellular responses is reflected by the fact that patients with several different PIDs with hereditary impairments in the adaptive and innate immune system, including SCID and CID with missense mutation in *IL2RG* (4, 5), loss-of-function mutations in DOCK2 (6) and DOCK8 (7), defects in intrinsic and innate immunity with loss-of function mutations in TYK2 (8), IFNGR1 (9), RTEL1 (10, 11), monogenic or digenic deficiencies in POLR3A and POLR3C (12), and haploinsufficiency in GATA2 (13, 14) are at risk of developing severe VZV-infection, while those with functional defects in granulocytes and those with pure agammaglobulinaemia are not.

Hemophagocytic lymphohistiocytosis (HLH) is a hyperinflammatory syndrome diagnosed following a molecular diagnosis of primary HLH or when five out of eight clinical and laboratory criteria are met (15). In addition to familial hemophagocytic lymphohistiocytosis and X-linked lymphoproliferative syndrome (XLP), HLH has been described in association with a variety of PIDs, and the clinical presentation of some of these cases has recently been summarized (16, 17).

Here, we describe two unrelated patients with GATA2-haploinsufficiency who developed severe primary VZV infection together with an HLH-like disease. In addition, we describe the immunological phenotype of these two patients compared with healthy controls and with two other patients with GATA2-haploinsufficiency without VZV infection.

## Case Presentations

**Figure 1:**
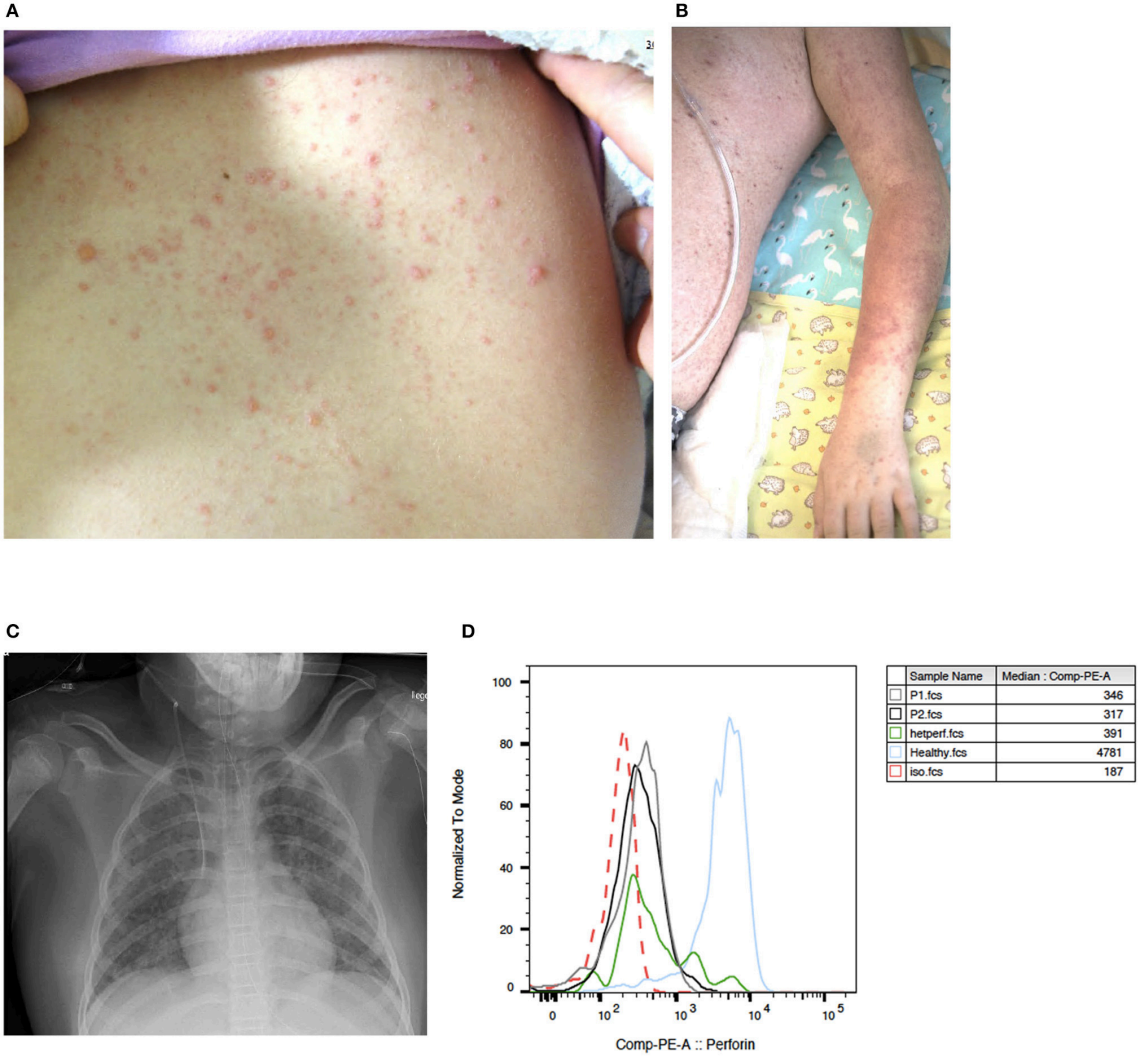
Clinical presentation of VZV disease in GATA2-haploinsufficient patient **(A)** An erytematous, vesicular skin rash on the back of patient 1 with GATA2-haploinsufficiency, 4 days after the onset of VZV disease. **(B)** Targetoid lesions on the left arm of the same patient, 7 days after the onset of illness. **(C)** A chest X-ray 8 days after the onset of illness, showing diffuse alveolar involvement with a miliary pattern of pulmonary opacities. **(D)** Histograms showing perforin expression in CD56+CD16+ lymphocytes from patient 1 (gray), patient 2 (black), an individual with heterozygous A91V PRF1 mutation (green), a healthy control (blue) and isotype control (red dotted line) as assessed by intracellular flow cytometry.

**Patient 1** is an 8-year-old girl who first presented with abdominal pain, an erythematous, vesicular skin rash (Figure 1A) and subfebrile body temperatures over a 4-day period. In the preceding month, the patient had been referred for a medical workup for persistent warts on the hands and feet. At that time, recurrent furuncles been noted. On admission, the serum AST level was 490 U/l (normal range < 48), the ALT was 374 U/l (normal range < 39), and the LDH was 1619 U/L (normal range < 236). The patient's blood count and prothrombin time (PT), activated Partial Thromboplastin Time (aPTT), bilirubin, fibrinogen, and creatinine levels were all normal. Although the skin rash was not classic for VZV and could thus not be unambiguously assigned to VZV infection, empiric treatment with acyclovir was initiated immediately. A biopsy showed non-specific changes with spongiosis of the epidermis, perivascular lymphocytic infiltrates and rare eosinophilic granulocytes. The patient's mother reported that the patient had chicken pox some years before admission. However, we could not detect anti-VZV antibodies in a serum sample taken 1 month before admission. Varicella zoster virus DNA was detected in the patient's blood (peaking at 537,000 copies/ml) and in the fluid from skin vesicles. HSV-1 and−2 PCR of vesicle fluid as well as HHV6, EBV, and CMV PCR of blood were negative. Mild hypogammaglobulinemia was noted (IgG 6.5 g/l; normal range: 7.6 – 14.5) and intravenous immunoglobulins (0.4 g/kg) were administered on day 2 and 4 as a supportive treatment. Seven days after the onset of illness, the skin rash progressed to targetoid lesions (Figure 1B) and the patient developed fever and tachy-dyspnoea. Respiratory insufficiency on day 8 after the onset of illness required mechanical ventilation. A miliary pattern of pulmonary opacities was noted on a chest X-ray (Figure 1C). The patient's laboratory parameters gradually deteriorated with progression to bicytopenia. On day 13, specific laboratory findings were noted: anemia (hemoglobin: 73 g/l), neutropenia (absolute neutrophil count: 710 cells/μL), elevated ferritin (peak value: 7090 μg/L; normal range 7 – 69), elevated triglycerides (peak value: 5.8 mmol/L; normal range 0.5 – 2.2), elevated soluble IL-2 receptor (peak value: 1622 pg/ml; normal range < 477), documentation of haemophagocytosis in a bone marrow aspirate; an elevated platelet count, and elevated fibrinogen. The spleen was not enlarged. Overall, the patient's clinical and laboratory profile met the diagnostic criteria for HLH (Table 1), and so treatment with corticosteroids (1.5 mg/kg/d) was initiated on day 13. The patient progressively recovered from hepatitis, pneumonitis and HLH. Leukocyte subpopulations, which had contracted during the VZV illness expanded again thereafter (Supplementary Table 1). The rash resolved after having progressed from vesicles to scabbing. Acyclovir was discontinued after 21 days, and corticosteroids were tapered over a total treatment period of 4 weeks.

**Table 1:** HLH features of patient 1 and 2.

**HLH criteria:**		**Patient 1**	**Patient 2**
1.	Fever ≥38.5	**Yes**	**Yes**
2.	Splenomegaly (palpable below costal margin or increased size by imagery)	**No**	**Yes**
3.	Cytopenias (affecting ≥2 out of the 3 lineages): – Hemoglobin (< 90 g/l) – Neutrophilic granulocytes (< 1.0 × 10^9^/l) – Platelet count (< 100 × 10^9^/l)	**Yes:** 73 g/l 0.71 × 10^9^/l (Plts: not fulfilled)	**Yes:** (Hb: not fulfilled) 0.38 × 10^9^/l 53 × 10^9^/l
4.	Hemophagocytosis (in bone marrow or CSF)	**Yes**	NA
5.	Hyperferritinemia (≥500 μg/l)	**Yes:** 7,090 μg/l	**Yes:** 4,510 μg/l
6.	Hypertriglyceridemia (fasting level: ≥3.0 mmol/l) or hypofibrinogenemia ( ≤ 1.5 g/l)	**Yes:** triglyceride fasting level: 5.8 mmol/l	NA
7.	Increased soluble CD25 (≥2,400 U/l)	**No** (although above normal level)	**No**
8.	Decreased protein expression (Perforin, SAP, or XIAP) or deficient NK-cell degranulation	Perforin expression in lower range of normal, NK cell degranulation on the threshold	Perforin expression in lower range of normal, NK cell degranulation on the threshold
Totally fulfilled criteria^*^ **(**≥ **5 out of 8)**		**Criteria fulfilled** (5 out of 8 fulfilled)	**Criteria not fulfilled, not all analyzed** (4 out of 6 fulfilled)

* *Diagnosis of an active HLH episode can be established if 5 out of the 8 criteria listed above are fulfilled. NA, not analyzed*.

A history of persistently low cell counts of neutrophilic granulocytes and B cells, hypogammaglobulinaemia, and increased susceptibility to infections (furunculosis and persistent warts) had prompted us to sequence the patient's *GATA2* gene. This confirmed the presence of a heterozygous mutation (c.1172_1175del, p.E391Gfs^*^85) compatible with GATA2-haploinsufficiency.

More than 1 year later, the patient again developed bicytopenia, hyperferritinaemia, hypertriglyceridaemia, hypofibrinogenaemia, an elevated soluble IL-2 receptor level, and transaminitis after an episode of fever of unknown origin. Treatment with corticosteroids (2 mg/kg/day) and a broad-spectrum antibiotic was initiated, and the patient recovered. No pathogen could be identified.

**Patient 2** is a 7-year-old boy with known GATA2-haploinsufficiency who presented with fever, skin rash, and cough. Three years earlier, the patient had been treated for recurrent fever, oral aphthosis, and recurrent furunculosis. Together with the observed lymphopenia (affecting CD4 T cells, B cells, and NK cells) and the presence of treatment-resistant warts on the patient's mother's hands, these immunological findings prompted us to sequence *GATA2* in the patient and his mother. The diagnosis of GATA2-haploinsufficiency was confirmed by the presence of a c.(16bp tandem repeat in exon 4), p.T347fs) mutation. On admission, the boy was in good general condition. He showed mild fever (38.5°C), hepatosplenomegaly and an erythematous, vesicular skin rash suggestive of chickenpox, prompting empiric intravenous treatment with acyclovir. Mild hypogammaglobulinemia was known in this patient (IgG 6.1 g/l; normal range: 6.7 – 12.1) but was not substituted before. After admission, varicella immunoglobulins (22 IU/kg) and intravenous immunoglobulins (0.4 g/kg) were administred on day 0 and 2, respectively. Diagnosis was confirmed by positive VZV PCR in samples from skin lesions and peripheral blood (peak value: 183,572 copies/ml). CMV PCR of blood was negative. Low EBV DNA load had been detected in this patient 1 month before VZV-infection and EBV DNA load slightly increased during VZV-infection (max 424 copies/ml, normal range < 100) and became negative 2 months thereafter. On day 2, the patient developed pancytopenia (hemoglobin: 95 g/L; absolute neutrophil count: 380 cells/μL; platelet count: 53 G/L). On days 2 and 3, specific laboratory findings were noted: an elevation in serum ferritin (from 820 to 4,510 μg/L within 3 days) and triglycerides (from 0.6 to 2.2 mmol/l within 3 days). The clinical signs and laboratory results were consistent with a diagnosis of developing HLH (Table 1), and so treatment with corticosteroids (2 mg/kg/d) was initiated.

Natural killer cell function assays revealed a slight reduction in degranulation (as assessed by CD107a expression) in both patients (9.4% in patient 1 and 8.8% in patient 2; normal range >10%) upon stimulation with K562 target cells. Perforin expression in both GATA2-haploinsufficient patients was within the lower range compared to an adult control and within the range of a control individual with known heterozygous A91V-perforin-mutation. SAP and XIAP expression in patient 2 were normal (Figure 1D).

**Figure 2:**
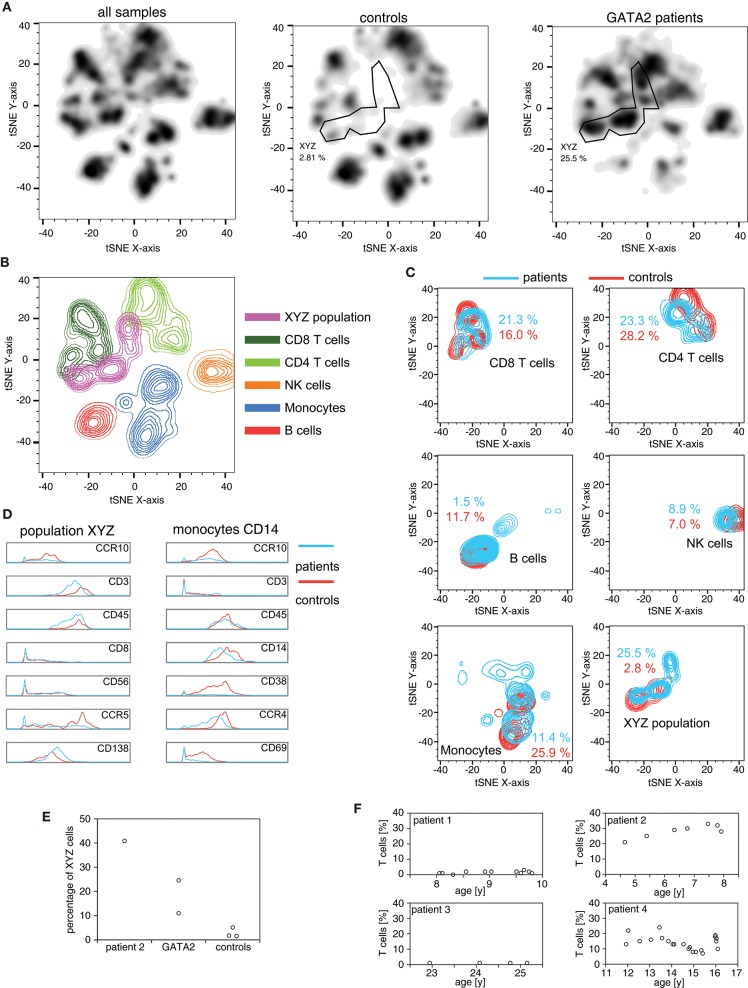
High-dimensional mass cytometry for the analysis of immune populations in GATA2-haploinsufficient patients PBMCs of 3 patients (patients 2–4) and 3 healthy controls were stained with 27 heavy-metal labeled antibodies (Supplementary Table 2) and acquired on a CYTOF2 mass cytometer. **(A)** Cells identified as CD45+ were exported, downsampled to 900 events and the combined dataset (5,400 cells) subjected to dimensionality reduction using the t-SNE algorithm. Density plot of total (left panel), control (middle panel) and patient (right panel) cells based on tSNE analysis. The manually gated XYZ population consist of closely grouped cells present mainly in the patients and not belonging to any immune subpopulation defined by manual annotation. **(B)** Color-coded manual gates are plotted by density for the combined dataset. **(C)** Density plots of the different subpopulations in patients and controls with the fraction out of all CD45+ cells indicated. **(D)** Histograms of markers with the largest expression discrepancies between patients and controls in the XYZ population and among monocytes based on visual analysis. **(E)** Percentages of XYZ cells from the CyTOF data, out of all CD45+ plotted for patient 2 (HLH), the 2 additional patients with germline *GATA2*-mutations (patient 3 and 4) and the 3 healthy controls. **(F)** Timecourse of TCRγδ+ cells (percentage of lymphocytes) measured by conventional flow cytometry in GATA2-haploinsufficient patients 1 to 4.

Five months before the VZV episode, patient 2 was included, together with two other patients with GATA2-haploinsufficiency (patients 3 and 4), in a pilot study aimed at characterizing the immunological phenotype of this disease using mass cytometry (CyTOF). Patient 3 is the mother of patient 2 with an identical genotype (p.T347fs). In patient 4, a mutation in *GATA2* c.593delC was found, which is predicted to result in p.Ala198fs with a premature stop codon 19 positions downstream. To visualize the lymphocyte pattern differences in peripheral blood samples from these three patients and three healthy controls, we used t-Disturbed Stochastic Neighbor Embedding (tSNE) to visualize mass cytometry datasets (Figures 2A,B), using the antibody panel shown in Supplementary Table 2. All patients with GATA2-haploinsufficiency had markedly reduced peripheral blood B cells, NK cells and monocytes (Figure 2C). The patients furthermore displayed an exceedingly high frequency of a CD3+ CD56+ CD16- CD8- CD4- cell population (“XYZ-population” in Figures 2A,D,E). Although largely different in ratios, this and the monocyte population retained the same phenotype as the ones present in healthy controls (Figure 2D). A large proportion of the CD3+ CD56+ CD16- CD8- CD4- cell population phenotypically corresponded to TCRγδ+ or NKT lymphocytes. This was confirmed by flow cytometry analysis of samples collected at different time points using a TCRγδ specific antibody (Figure 2F). A marked accumulation (20% to 30% of lymphocytes) of these TCRγδ+ cells was noted between the age of 4 and 8 years in patient 2. Patient 4 also showed an elevated percentage of these cells (10% to 20% of lymphocytes between the age of 12 and 16 years). On the contrary, TCRγδ+ cells in patient 1 and patient 3 remained below 5% of lymphocytes during the period of monitoring (between the age of 8 and 10 years for patient 1, and 22 and 25 years for patient 3), raising the possibility that populations other than TCRγδ+ T cells are expanded in GATA2-haploinsufficiency (Figure 2F).

## Discussion

Here we describe two patients with GATA2-haploinsufficiency with severe VZV infection and HLH-like disease. Although increased susceptibility to VZV is regularly seen in patients with GATA2-haploinsufficiency, the combination of GATA2-haploinsufficiency and HLH-like disease has not been described so far.

Features consistent with HLH included fever, cytopenia involving at least two cell lineages, and elevated serum ferritin (>4,500 μg/L). Haemophagocytosis in the bone marrow aspirate and elevated serum levels of triglycerides and of soluble IL-2R in one of the patients are additional characteristics suggestive of HLH. Of note, patients with GATA2-haploinsufficiency may have baseline cytopenias, as this was the case in patient 2. Diagnosis of HLH in patients with baseline cytopenias must be made carefully with special attention on the dynamics of clinical course and laboratory parameters to ensure early recognition. Both patients were successfully treated for VZV and HLH, and recovered without sequelae. Although the occurrence of HLH has been reported in a variety of PIDs (5, 6), the combination of GATA2-haploinsufficiency and HLH has, to the best of our knowledge, only been described twice in the context of herpes virus infection: once in a patient with herpes simplex virus infection (18), and once in a patient with marked EBV viremia and an EBV-driven T cell lymphoproliferative disease (19), but not with VZV. In one of our patients, the HLH-like syndrome was already present when the PID was diagnosed. GATA2-haploinsufficiency is therefore a potential differential diagnosis in patients presenting with the clinical features of HLH – especially in cases of persisting cytopenia after recovery from HLH.

In the two patients with GATA2-haploinsufficiency described here, VZV infection had probably triggered the HLH-like syndrome. Interestingly, the medical history of patient 1 was suggestive of prior VZV primary infection but no VZV-specific antibodies were detected in serum taken before the HLH-eliciting VZV infection. Patients with GATA2-haploinsufficiency are known to be at risk for unusual or more severe herpes virus infections (2, 20), but the precise mechanism has yet to be defined. Severe VZV-infection was part of the presentation of one of the earliest reported patients reported by Biron et al. (13), subsequently characterized as GATA2-haploinsufficient patient (14). Although VZV can trigger HLH in patients under immunosuppression or in otherwise healthy individuals, the literature, however, contains, to the best of our knowledge, no reports on VZV-triggered HLH in PIDs (16, 17, 21). Perturbation of the immune homeostasis between viral control and immune activation is thought to be an important factor in the viral triggering of HLH. This hypothesis is supported by data from murine models of HLH, where CD8 T cells with genetic defects in cytotoxic activity and interferon gamma have a pivotal role in the pathogenesis of an HLH-like condition (22, 23). Although NK cell depletion does not prevent the development of HLH (22), NK cells were shown to control T cells via a perforin-dependent mechanism (24, 25, 26). Altered cytotoxic granule content associated with adaptive NK cell differentiation has been previously shown in GATA2-haploinsufficient patient (27). In accordance with these findings, patient 1 and 2 showed low perforin expression. Thus, NK cell deficiency might not only predispose to a susceptibility to infections but might have accentuated the susceptibility to HLH in our GATA2-haploinsufficient patients due to a deficiency in an important immunoregulatory role of NK cells.

Our analysis of the immunological repertoire in GATA2-haploinsufficient patients shows that there is an unexpected expansion of CD3+ CD56+ CD16- CD8- CD4- cells. Using flow cytometry, we confirm a high proportion of TCRγδ+ cells in peripheral blood of patient 2. The normal proportion of TCRγδ+ cells in patient 1 confirms that HLH can occur without TCRγδ+ cell expansion. Also, low percentage of TCRγδ+ T lymphocytes in patient 3, together with the evidence of expanded CD3+ CD56+ CD16- CD8- CD4- cells, raises the possibility that other cell types, like NKT cells, can expand in GATA-2 deficiencies. The cause behind the expansion in TCRγδ+ cells in two of the four GATA2-haploinsufficient patients is unknown.

GATA2-haploinsufficiency is associated with poor maturation and low numbers of peripheral blood NK cells (also noted in our patients before VZV infection). Slightly reduced NK cell degranulation was observed in both GATA2-haploinsufficient patients with HLH. This is consistent with a previous report of decreased NK cell cytotoxicity in a GATA2-haploinsufficient patient (18). It is tempting to speculate that deficiency in NK cell number and function disturbs the immunoregulatory role of NK cells and makes GATA2-haploinsufficient patients more prone to HLH-like disease. HLH-like disease might, therefore, reflect not only impaired infection control but also a genetic predisposition to hyperinflammation.

Since GATA-2 haploinsufficiency is characterized by monocytopenia, together with B, NK and DC lymphopenia, discovering a population expanded in GATA-2 patients is remarkable. Because the phenotype of this T cells resembles that of TCRγδ+ T lymphocytes and NKT cells, we are suggesting that this could be the hyper-responsive population driving HLH symptoms. The relevance of this population, or any other, in causing HLH is unclear but the innate-like phenotype of TCRγδ+ T lymphocytes and NKT cells, with their capacity of secreting large amounts of inflammatory cytokines, is suggestive that these populations could escalate a viral infection to an HLH like episode.

The present report thus expands the clinical spectrum of GATA2-haploinsufficiency and highlights new aspects of immune dysregulation in this disorder. However, the mechanisms leading to HLH-like disease have yet to be fully understood.

## Ethics Statement

Written informed consent was obtained from all study participants. The study was approved by the local ethics' committee (KEK-ZH-Nr. 2015-0555).

## Author Contributions

SP initiated the study, revised the manuscript and was involved in patient care. MF revised the manuscript and was involved in patient care. BV conducted NK testing and CyTOF and contributed to writing the manuscript. JT, AS-B, MT and LW were involved in patient care and revised the manuscript. SH performed genetic testing, gave consultative support in patients' care and revised the manuscript. KS performed genetic testing. SV contributed to data analysis and revised the manuscript. JP was involved in patient care and wrote the manuscript.

### Conflict of Interest Statement

The authors declare that the research was conducted in the absence of any commercial or financial relationships that could be construed as a potential conflict of interest.
