# Supplementary material for: Life-Threatening Primary Varicella Zoster Virus Infection With Hemophagocytic Lymphohistiocytosis-Like Disease in GATA2 Haploinsufficiency Accompanied by Expansion of Double Negative T-Lymphocytes
Source: Front Immunol. 2018 Dec 3;9:2766. doi: 10.3389/fimmu.2018.02766 (PMC6289061; doi:10.3389/fimmu.2018.02766)
Supplement: Supplementary file 1 [file Table_1.docx]

Supplemental material to:

# Life-threatening primary varicella zoster virus infection with

# haemophagocytic lymphohistiocytosis-like disease in GATA2 haploinsufficiency accompanied by

# expansion of double negative T-lymphocytes

**Authors:** Seraina Prader^1^, MD, Matthias Felber^1,2^, MD, Benjamin Volkmer^1^, PhD, Johannes Trück^1^, MD, DPhil, Agnes Schwieger^3^, MD, Martin Theiler^3,4^, MD, Lisa Weibel^3,4^, MD, Sophie Hambleton^5^, MD, PhD, Katja Seipel^6^, PhD, Stefano Vavassori^1^, PhD, Jana Pachlopnik Schmid^1,7^, MD, PhD.

**Affiliations:**

^1^ Division of Immunology, University Children's Hospital Zurich, Zurich, Switzerland

^2^ Division of Stem Cell Transplantation, University Children's Hospital Zurich, Zurich, Switzerland

^3^ Department of Pediatric Dermatology, University Children's Hospital Zurich, Zurich, Switzerland

^4^ Department of Dermatology, University Hospital Zurich, Zurich, Switzerland

^5^ Institute of Cellular Medicine, International Centre for Life, Newcastle University, Newcastle upon Tyne, United Kingdom.

^6^ Department for Biomedical Research, University of Bern, Bern, Switzerland.

^7^ Pediatric Immunology, University of Zurich, Zurich, Switzerland

**Supplemental table 1. Leukocyte subpopulations before, during and after VZV infection in patient 1 and 2**

|  |  | **Patient 1** |  | **empty** |  | **Patient 2** |  |
| --- | --- | --- | --- | --- | --- | --- | --- |
|  |  |  |  |  |  |  |  |
|  | **Before varicella infection**  **(- 3 months)** | **During varicella infection** | **After varicella infection**  **(+3 months)** |  | **Before varicella infection**  **(-3 weeks)** | **During varicella infection** | **After varicella infection**  **(+1 month)** |
| IgA (g/l) | 0.61 | nd | 0.58 |  | 1.21 | nd | 1.11 |
| IgM (g/l) | 1.13 | nd | 0.72 |  | 1.15 | nd | 1.29 |
| IgG (g/l) | 6.17 | 6.52 | 14.5 |  | 6.09 | nd | 7.67 |
| IgG1 (g/l) | nd | nd | 12.2 |  | nd | nd | 4.79 |
| IgG2 (g/l) | nd | nd | 1.17 |  | nd | nd | 1.12 |
| IgG3 (g/l) | nd | nd | 1.34 |  | nd | nd | 0.29 |
| IgG4 (g/l) | nd | nd | <0.07 |  | nd | nd | <0.07 |
| Tetanus IgG (U/ml) | 577 | nd | 377 |  | nd | nd | nd |
| HiB IgG (µg/ml) | 3.14 | nd | 5.14 |  | nd | nd | nd |
| Leukocytes (x 10^9^/l) | 2.8 | 2.67 | 6.78 |  | 2.39 | 1.3 | 1.63 |
| Lymphocytes (x 10^9^/l) | 1.31 | 1.34 | 2.32 |  | 0.88 | 0.38 | 1.12 |
| TCR alpha/beta (x 10^9^/l) | 1.17 | 1.31 | 2 |  | 0.55 | nd | 0.75 |
| TCR gamma/delta  (x 10^9^/l) | 0.01 | 0 | 0.05 |  | 0.28 | nd | 0.31 |
| DNT* | 13.3% | 1.6% | 11.2% |  | 20.9% | 22.5% | 20.4% |
| CD4^+^ (x 10^9^/l) | 0.68 | 0.86 | 0.83 |  | 0.24 | 0.1 | 0.34 |
| CD4^+^CD45RA^+^ (x 10^9^/l) | 0.46 | 0.61 | 0.64 |  | 0.15 | nd | 0.22 |
| CD31^+^CD45RA/CD4^+^ (x 10^9^/l) | 0.22 | 0.31 | 0.35 |  | 0.06 | nd | 0.07 |
| CD8^+^ (x 10^9^/l) | 0.48 | 0.46 | 1.18 |  | 0.46 | 0.21 | 0.55 |
| CD19^+^ (x 10^9^/l) | 0.05 | 0.01 | 0 |  | 0.03 | 0.02 | 0.02 |
| CD20^+^ (x 10^9^/l) | 0.04 | 0 | 0 |  | 0.03 | 0.01 | 0.01 |
| CD16^+^CD56^+^ (x 10^9^/l) | 0.07 | 0 | 0.26 |  | 0.01 | 0 | 0.01 |
| CD14^+^ (x 10^9^/l) | 0.04 | 0 | 0 |  | 0.02 | nd | nd |

*DNT: % of double negative T cells

### Supplemental table 2. Antibodies used for Mass Cytometry

| **Target** | **Clone** |
| --- | --- |
| CD196/CCR6 | G034E3 |
| CD19 | HIB19 |
| CD127 | A019D5 |
| CD11b | ICRF44 |
| CD4 | RPA-T4 |
| CD8a | RPA-T8 |
| CD16 | 3G8 |
| CD25 (IL-2R) | 2A3 |
| CD138 | DL-101 |
| CD192 (CCR2) | K036C2 |
| CD45 | HI30 |
| CD279/PD-1 | EH12.2H7 |
| CD195/CCR5 | NP-6G4 |
| CD194/CCR4 | 205410 |
| CCR7 | G043H7 |
| CD28 | CD28.2 |
| CD69 | FN50 |
| CD294/CRTH2 | BM16 |
| CCR10 | 314305 |
| CD27 | O323 |
| CD45RA | HI100 |
| CD3 | UCHT1 |
| CXCR5 | RF8B2 |
| CD38 | HIT2 |
| HLA-DR | L243 |
| CD14 | M5E2 |
| CD56 | NCAM16.2 |
| CD45 | HI30 |
